# Persistent Organic Pollutants: Not Finished Yet

**DOI:** 10.1289/ehp.116-a199a

**Published:** 2008-05

**Authors:** Carol Potera

Although polychlorinated biphenyls (PCBs) were banned in the United States in 1977, they persist in the environment. These bioaccumulative chemicals can damage the immune, reproductive, endocrine, and nervous systems. When looking for environmental sources of PCBs, scientists generally measure dietary intake from fish, meat, and milk. However, older wood floor finishes that harbor PCBs may present an underestimated route for exposure, finds Ruthann Rudel, a toxicologist at the nonprofit Silent Spring Institute in Newton, Massachusetts.

Animal studies show that PCBs decrease the size of the thymus gland and suppress antibody production and immune responses. People exposed to PCBs can suffer from skin rashes, changes in blood and urine that can indicate liver damage, irregular menstrual cycle, lowered immune response, fatigue, and headache. In children, poor cognitive development is reported. The International Agency for Research on Cancer classifies PCBs as probable human carcinogens.

From 1999 to 2001, Rudel and her colleagues monitored 89 indoor pollutants linked to breast cancer in 120 homes on Cape Cod, where breast cancer rates are among the highest in Massachusetts. As reported 13 September 2003 in *Environmental Science & Technology*, detectable levels of PCBs were found in about a third of the residences, and two houses had exceedingly high levels of PCBs in air and dust.

Five years later, Rudel revisited these two houses to measure air and dust concentrations of the congeners PCB52, PCB105, and PCB153 as well as occupants’ blood levels of 10 congeners measured in 1999–2002 as part of the National Health and Nutrition Examination Survey (NHANES). Air and dust levels remained exceptionally high, with dust values ranging from 21 to 190 μg/g—up to 20 times higher than reported in other studies of indoor PCB exposure. Three of the occupants, all of whom had lived in their homes for more than a decade, showed elevated blood levels of PCBs as high as 1,520 ng/g, placing them in the top 5% of people living in the United States. A fourth occupant, who had lived in her house for only six months, had a blood level of 179 ng/g. The study is published as volume 7, article 2 (2008) of the online journal *Environmental Health*.

None of the occupants ate enough fish to account for the high blood levels of PCBs. “We were really stumped,” says Rudel. Then the homeowner with the highest PCB levels recalled that 50 years earlier he had finished his wood floors with high-gloss “Fabulon” varnish. In the 1950s and 1960s, Fabulon was a popular coating sold nationwide to give floors a “bowling-alley shine.” A now-discontinued reference manual, *Clinical Toxicology of Commercial Products*, confirmed that Fabulon contained PCBs between 1950 and 1969. Before being banned, PCBs were not only added to varnishes but also to caulk, paint, linoleum, and ceiling tiles, where they served as stabilizers, sealants, and adhesives.

The same homeowner had sanded one of his floors just before Rudel returned to collect samples for the latest study. However, the floors in the other house had not been refinished in years. Rudel suspects, but has not confirmed, that the floors may be giving off PCBs, with sanding releasing even higher levels of dust packed with PCBs. She hopes to conduct further research—such as whether a peak time for exposure occurs when sanding off PCB-laced finishes, or whether carpets later laid over finished wood floors limit airborne exposure—that might shed light on safer ways to remove PCBs.

What can homeowners do, pending further information? “I’m not happy about how little I can tell people,” Rudel admits. Foremost, she advises not sanding old wood floors. PCB analysis is expensive, and Rudel knows of no laboratories that service homeowners. However, some floor-finishing businesses offer a dustless sanding process that pumps toxic dust into outdoor tanks.

“Rudel certainly found an unrecognized source of PCBs that underscores the importance of inhalation as an underappreciated but important source of exposure,” says David Carpenter, a public health physician at the University at Albany, State University of New York. The results add to a growing body of awareness that building materials can be a significant source of PCBs indoors. “The assumption that PCBs stay put simply is not true,” Carpenter says.

## Figures and Tables

**Figure:**
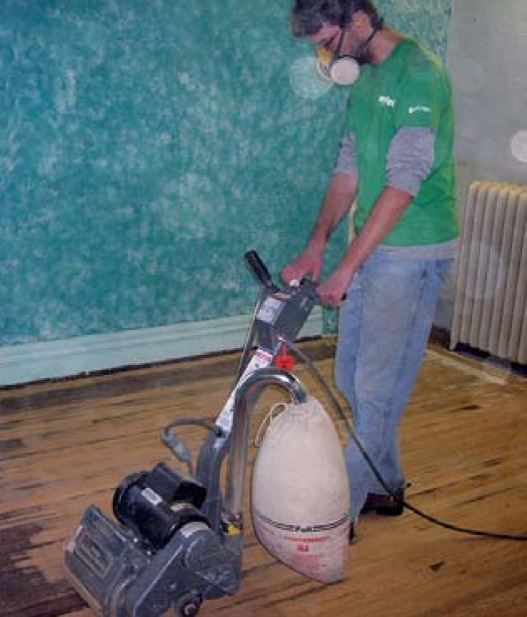
Old wood floors may be a source of indoor PCB exposure

